# The effects of rumen-protected tryptophan (RPT) on production performance and relevant hormones of dairy cows

**DOI:** 10.7717/peerj.13831

**Published:** 2022-09-13

**Authors:** Hui Ma, Songyang Yao, Libing Bai, Sarvvl Bai, Guoshi Liu

**Affiliations:** 1 Beijing Sanyuan Breeding Technology Co., Ltd., Beijing, China; 2 Beijing Key Laboratory of Animal Genetic Improvement, Key Laboratory of Animal Genetics and Breeding of the Ministry of Agriculture, National Engineering Laboratory for Animal Breeding, College of Animal Science and Technology, China Agricultural University, Beijing, China; 3 Beijing Sunlon Livestock Development Co. Ltd., Beijing, China

**Keywords:** RPT, Melatonin, Milk composition, Hormone

## Abstract

Tryptophan is an essential amino acid that cannot be synthesized in mammals. Therefore, the dietary supply of tryptophan is critical for the health and production performance (*e.g.*, milk) of mammals. In the present study, 36 lactating Holstein cows were used, of which 24 cows were in the rumen-protected tryptophan (RPT) feeding groups with different doses at 14 g/d and 28 g/d, respectively and 12 cows were in the control group. This approach could avoid dietary tryptophan being degraded by the rumen microorganisms and improve its bioavailability for cows. The results showed that RPT increased milk protein percentage, milk protein yield, milk solid non-fat (SNF), and milk yield. In response to RPT treatment, the levels of melatonin (MT), prolactin (PRL), and insulin-like growth factor-1 (IGF-1) were significantly increased in the serum of cows compared to the controls. RPT feeding improved nutrient utilization efficiency and lactation performance of dairy cows, which enhanced the quality of milk.

## Introduction

Tryptophan (TRP) is an essential amino acid that mammals have lost the ability to synthesize it during evolution. Most animals obtain TRP from their diets. Thus, it is widely used as a food supplement in agricultural animals (Yao et al., 2011). Ruminants, including cows, in most of the cases were unable to obtain adequate tryptophan from their diets (McLaren, Anderson & Barth, 1965; Kollmann et al., 2008). The reason is that the ruminant’s feed protein often lacks tryptophan, and thus the common feeding materials usually cannot provide sufficient TRP to ruminants for their maximum requirement (Li & Song, 2020). Another reason is that large quantity of the supplemented dilatory tryptophan are degraded by rumen microorganisms (Tan et al., 2021).

TRP plays an important role in animal health including their feeding behavior, growth, reproduction, immunity, antistress activity, and other functions (Wu, 2010). It has been reported that tryptophan deficiency leads to nitrogen deposition reduction, growth retardation, low feed intake, stress sensitivity, and rough hair (Henry et al., 1992; Coşkun et al., 2006; Cubero et al., 2006). Dairy cows mainly acquire amino acids by digesting proteins from microbes and plants. Quality and quantity of the absorbed amino acids are critical for the quality and quantity of milk produced by dairy cows. The essential amino acid, TRP, has several unique characteristics. Tryptophan is the precursor for several neurotransmitters and metabolic regulators. For example, tryptophan can be metabolized to several bioactive molecules, including serotonin (5-hydroxytryptamine, 5-HT), melatonin (MT), and niacin (Moehn, Pencharz & Ball, 2012). Among them, serotonin was a key factor for calcium homeostasis, modulating calcium concentration in blood, which affected the parturient calcium homeostasis in dairy cows (Hernández-Castellano et al., 2017). A study showed that supplementing with L-Tryptophan increased medium protein and alters expression of genes and proteins involved in milk protein synthesis and energy metabolism in bovine mammary cells (Conejos et al., 2021). Supplementation of L-Tryptophan’s derivative, melatonin, improves milk composition (Yao et al., 2020). Milk yield depends on mammary development and lactation functions, which are mainly regulated by reproductive hormones, such as prolactin (PRL), growth hormone (GH), and insulin like growth factor-1 (IGF-1) (Jørgensen, Knigge & Warberg, 1992; Farris et al., 1998; McMahon et al., 2001). Interestingly, tryptophan supplementation increased reproduction performance, milk yield, and milk composition in lactating sows and growth performance of their piglets. As we realize that ruminants have the different digestive system from the sows. The tryptophan supplemented in the diet will be significantly degraded by the microorganisms in the rumen before it enters the blood of the ruminants.

Thus, in this study, the rumen-protected tryptophan was formulated by high temperature modulation and strong extrusion by granulating machine and coated by other composition in order to reduce their degradation in the rumen. Therefore, we expected that this process would increase the bioavailability of tryptophan supplementation in dairy cows. Accordingly, the serum levels of hormones which related to the milk production including MT, PRL, GH, and IGF-1 would be measured. In addition, the milk yield, milk composition would also be analyzed after RPT administration. The results would provide valuable information on whether the RPT could increase the efficiency of tryptophan supplementation in ruminants, particularly in dairy cows.

## Materials and Methods

### Animals

Thirty-six healthy Holstein cows were selected in August for this study from Beijing Sunlon Livestock Development Co. Ltd. Before the study, general information for these cows was recorded, including body weight (693  ± 22.6 kg), lactation days (143  ± 11 d), milk yield (43.3  ± 8.1 kg/d), and fetal times (3.1  ± 0.8). At the end of the experiment, all experimental cows returned to normal production. The experimental site was in the Beijing Dairy Cow Center seed multiplication farm in the Yanqing District (longitude: 115.97°E, latitude: 40.47°N). The experiment was approved by the China Agricultural University Laboratory Animal Welfare and Animal Experimental Ethical Inspection Committee, and the animal experiment approval number was AW61301202-1-1.

### Experimental diet and feeding management

Experimental cows were raised in semi-enclosed cowsheds with 23∼25 °C (both water sprinkler system and fan were turned on to control the temperature) during the experiment. The cows were exposed to the natural light/ dark cycles without humans interfering. Each separated feeding trough was placed at neck clamp position. The time of feeding was at 7:00 and 16:00 daily. Each experimental cow was fed the same amount of feed without tie-stall. The time of milking was at 7:00, 14:00, and 19:30 daily. The experimental diet was prepared according to the National Research Council (NRC) (2001) Nutrient Requirements of Dairy Cattle. Dietary composition, nutrient values, energy, metabolizable protein, and tryptophan supply were listed in Table 1. The nutrient composition and content of the feed raw materials were listed in Table 2.

### Experimental design

Thirty-six cows were randomly divided into three groups: Twelve cows in the 14 g/d RPT feeding group, twelve cows in the 28 g/d RPT feeding group, and twelve cows in the control group. The control group had the same feeding process as RPT groups but without RPT. The RPT was purchased from Beijing Feeding Feed Science Technology Co., Beijing, China. The degradation rate of RPT in the rumen was 12%, and the rumen bypass ratio of RPT was 77.5% according to the results of preliminary experiments conducted by the manufacturer. RPT contained 55% TRP and 45% other composition (admixture, starch, dextrin, sodium carboxyformic acid celluloseand rumen fatty powder).

### Delivery method

Corn flour was mixed with water to form a paste. The RPT was sprinkled on the 20-gram sticky corn surface and fed to the cows every day during the experimental period. The control group was also fed with cornflour without RPT. This method not only avoided losses during feeding but also ensured that each cow received equivalent amounts of RPT. The cows were fed every day during the experimental period. The experimental period was 52 d, which included a preliminary trial period of 10 d and a trial period of 42 d. The corn flour without RPT was fed and the blood was collected in the preliminary trial period, which allowed cows to adapt to the feeding process and experimental environment.

**Table 1:** Ingredient composition (air-dry basis) and nutrient levels (DM basis) of diets.

**Ingredient**	**%**	**Nutrient level**	
Chinese wildrye hay	0.6	Dry matter (%)	56.8
Alfalfa hay	5.5	NEL (MJ/kg)	5.78
Corn silage	52.4	Crude protein (%)	17.9
Corn	19.3	EE (%)	2.67
Soybean meal	6.9	NDF (%)	45.5
Cottonseed meal	0.9	ADF (%)	17
Cottonseed	1.4	ASH (%)	7.06
Wheat bran	4.3	Calcium (%)	0.8
Cifen	5.7	Phosphorus (%)	0.4
Megalac protected fat	1.4	Tryptophan (%)	0.063
Dicalcium phosphate	0.5		
Sodium bicarbonate	0.6		
Salt	0.2		
Premix	0.3		
Total	100		

**Notes.**

Cifen is a byproduct of wheat powder, which is mainly composed of finely bran crumbs and part of wheat endosperm.

**Table 2:** The composition and content (DM basis) of ingredients.

**Ingredients**	**Nutrient level (%, DM)**					
	**DM (%)**	**CP (%)**	**NDF (%)**	**ADF (%)**	**ASH (%)**	**EE (%)**
Chinese wildrye hay	93.6	5.22	70.05	39.1	5.05	1.01
Alfalfa hay	91.85	15.05	42.9	17.14	8.23	1.12
Corn silage	31.7	9.53	46.99	27.09	5.02	2.11
Corn	90.52	7.75	14.67	2.57	1.37	2.78
Soybean meal	87.91	44.59	14.33	6.74	6.21	1.9
Cottonseed	89.89	21.2	53.25	28.21	4.34	7.87
Wheat bran	90.02	18.16	47.65	10.48	5.48	2.43
Cottonseed meal	89.64	42	45.11	23.64	6.5	2.22
Wheat middling	89.98	17.33	24.53	5.66	3.12	4.34

### Methods of sample collection and determination

#### Recording of feed intake

The feeds offered and leftovers of each cow were accurately recorded, and the dry matter intake of each cow was recorded and calculated.

#### Methods of milk samples collection

The milk samples were collected three times each day on day 0, day 7, day 14, day 21, day 28, day 35, and day 42 during the trial period, and milk yield (kg/cow/d) was recorded. The samples of the milk collected in the morning, afternoon, and evening were mixed at 40%, 30%, and 30% according to the milk yield in a day, respectively. Fifty-milliliter milk samples were collected from all trial cows at 10:00 and 22:00 on day 0, day 2, day 4, day 8, and day 16 after tryptophan supplementation. The 50 mL milk samples with preservatives were stored at 4 °C with preservative until the dairy herd improvement (DHI) test was performed within 7 days.

#### Dairy herd improvement (DHI) test

The somatic cell counts (SCC) was determined by Fossomatic™ FC (Serial No. 91755377, Part No. 60002326; Denmark), which was based on a flow cytometric method. Milk protein, fat, dry matter, and lactose were measured by MilkoScan FT+ (Serial No. 91755049, Part No. 60027086; Denmark), which was based on Fourier transform infrared spectrum analysis.

#### Methods of serum samples collection

Blood was collected from the coccygeal vessels from all trial cows at 10:00 and 22:00 on day 0, day 2, day 4, day 8, and day 16 after tryptophan supplementation. The serum samples were stored at −20 °C until use for detection of the levels of 5-HT, MT, PRL, IGF-1, and GH within a week.

The levels of 5-HT, MT, PRL, IGF-1, and GH in the serum samples were tested by radioimmunoassay (RIA) at the Beijing Huaying Biological Research Institute. The test kits were purchased from Tianjin Jiuding Medical Bioengineering Co., Ltd.

#### Statistical analyses

The data are expressed as the mean ± SEM (*n* = 12). One way analysis of variance (ANOVA) was used to analyze the normality of most of the samples, followed by Duncan’s multiple tests for comparison of the difference between the interested groups.

The blood and milk samples were collected repeatedly on different experimental days and at different times. Therefore, the repeated measures of MIXED (SAS Institute, version 9.1) were used. Experimental animals, different treatment groups, and different days were used as variables. The least-squares means were used for multiple comparisons. The contrasts (SAS Institute, Version 9.1) program analyzed the comparison between the control group and the trypto-phan addition group, including the intra-group comparison of the tryptophan addition group. A *p* < 0.05 was considered as a significant difference.

## Results

### The effects of RPT supplementation on milk yield

The results showed that at the 42 days after RPT supplementation the milk yield of RPT treated cows either in 14 g/d or in 28 g/d group was significantly increased compared to the control group (*n* = 12, *F* = 5.768, *p* < 0.05) (Table 3). However, no significant differences were observed in the dry matter intake (DMI) among the groups (*n* = 12, *p* > 0.05) (Table 4).

### The effects of RPT supplementation on milk composition

As shown in Table 5, the milk protein percentage (14 g/d: 3.41 ± 0.28% and 28 g/d: 3.42 ± 0.17% *vs* control: 3.04 ± 0.17%) (*n* = 12, *F* = 6.254, *p* = 0.011), milk protein yield (14 g/d: 1.56 ± 0.14 kg/d and 28 g/d: 1.56 ± 0.07 kg/d *vs* control: 1.24 ± 0.08 kg/d) (*n* = 12, *F* = 19.194, *p* < 0.0001), and SNF (14 g/d: 9.19 ± 0.17% and 28 g/d: 9.19 ± 0.22% *vs* control: 8.84 ± 0.25%) (*n* = 12, *F* = 5.379, *p* = 0.017) were significantly increased in RPT feeding groups compared to the control. There was no significant difference in the rest of the milk compositions among groups (*p* > 0.05) (Table 5).

**Table 3:** Effect of RPT supplementation on milk yield in dairy cows (kg/d).

**Experiment day**	**RPT level(g/d)**		
	**0**	**14**	**28**
0	44.9 ± 3.1	44.3 ± 3.3	44.6 ± 3.2
7	45.7 ± 3.3	44.2 ± 3.4	44.3 ± 4.0
14	41.8 ± 3.4	43.8 ± 3.6	42.8 ± 4.0
21	43.0 ± 3.5	44.1 ± 3.6	42.5 ± 3.9
28	44.8 ± 4.1	47.2 ± 3.9	43.4 ± 4.0
35	41.5 ± 3.5	43.6 ± 4.1	45.0 ± 4.0
42	40.3 ± 4.9	45.8 ± 4.2^**^	45.4 ± 4.3^**^

**Notes.**

The data are expressed as the mean ± SEM (*n* = 12). The least-squares means were used for multiple comparisons. At the same time, the contrasts (SAS Institute, Version 9.1) program analyzed the comparison between the control and RPT groups, including the intra-group comparison of the RPT group.

** *p* < 0.01 vs control (0) group.

**Table 4:** Effect of RPT supplementation on dry matter intake (DMI) in dairy cows (kg/d).

**Experiment day**	**RPT level (g/d)**		
	**0**	**14**	**28**
0	20.0 ± 0.9	20.7 ± 2.9	23.9 ± 1.9
7	23.8 ± 1.4	19.4 ± 1.7	20.3 ± 1.2
14	20.3 ± 1.1	21.9 ± 0.8	23.5 ± 3.6
21	20.8 ± 0.6	19.9 ± 2.3	18.0 ± 2.4
28	21.0 ± 1.8	21.6 ± 3.1	22.8 ± 1.3
35	17.7 ± 2.2	20.6 ± 1.7	18.3 ± 2.7
42	23.5 ± 2.8	24.7 ± 2.6	21.7 ± 2.3

**Notes.**

The data are expressed as the mean ± SEM (*n* = 12). The least-squares means were used for multiple comparisons. At the same time, the contrasts (SAS Institute, Version 9.1) program analyzed the comparison between the control and RPT groups, including the intra-group comparison of the RPT group. No significant difference has been detected among groups.

**Table 5:** Effect of RPT supplementation on milk composition in dairy cows during the experimental period.

**Milk composition**	**RPT level (g/d)**		
	**0**	**14**	**28**
Fat (%)	3.17 ± 0.39	3.58 ± 0.55	3.67 ± 0.50
Protein (%)	3.04 ± 0.17	3.41 ± 0.28^*^	3.42 ± 0.17^*^
Lactose (%)	4.87 ± 0.04	4.80 ± 0.07	4.80 ± 0.07
TS (%)	12.01 ± 0.49	12.70 ± 0.59	12.80 ± 0.60
SNF (%)	8.84 ± 0.25	9.19 ± 0.17^*^	9.19 ± 0.22^*^
Fat yield (kg/d)	1.26 ± 0.16	1.60 ± 0.26	1.68 ± 0.27
Protein yield (kg/d)	1.24 ± 0.08	1.56 ± 0.14^**^	1.56 ± 0.07^**^
Lactose yield (kg/d)	1.96 ± 0.17	2.18 ± 0.15	2.20 ± 0.19
TS yield (kg/d)	4.83 ± 0.38	5.77 ± 0.52	5.83 ± 0.51
SNF yield (kg/d)	3.56 ± 0.27	4.14 ± 0.36	4.17 ± 0.03
SCC (×10^3^cells/mL)	171.3 ± 46.7	146.1 ± 49.5	139.2 ± 56.5

**Notes.**

The data are expressed as the mean ± SEM (*n* = 12). The least-squares means were used for multiple comparisons. At the same time, the contrasts (SAS Institute, Version 9.1) program analyzed the comparison between the control and RPT groups, including the intra-group comparison of the RPT group.

* *p* < 0.05.

** *p* < 0.0001 vs control (0) group.

### The effects of RPT supplementation on serum hormone levels in dairy cows

#### 5-HT

**Figure 1:**
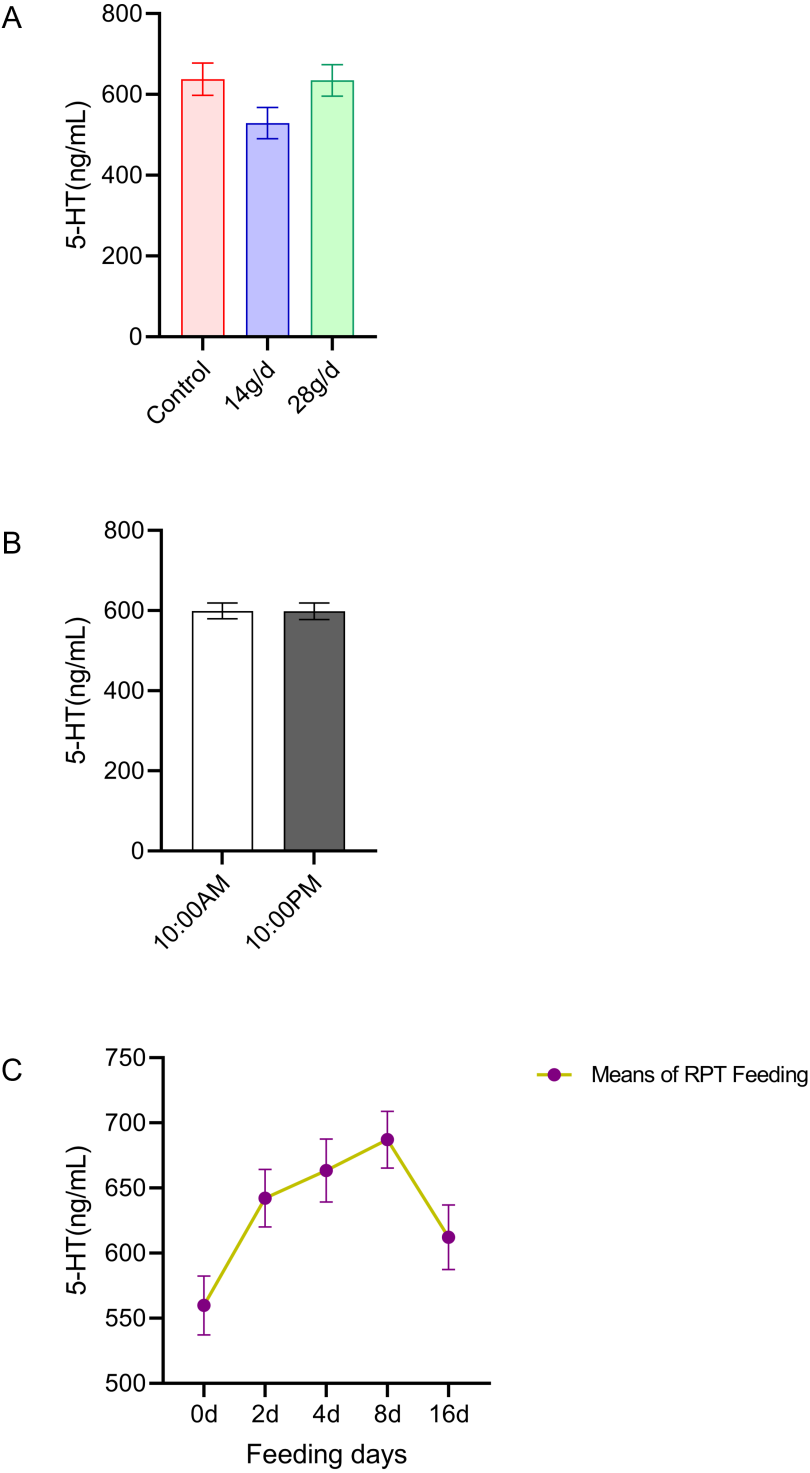
Effects of RPT feeding on 5-HT levels in serum. (A) 5-HT levels in different feeding groups; (B) 5-HT levels in different time after RPT feeding; (C) changes of 5-HT levels during RPT feeding days. The data are expressed as the mean ± SEM. One Way Analysis of variance (ANOVA) test was used to analyze the normality followed by student t-tests for comparison of the difference between the interested groups. For (B) only Student t-test was used.

The results indicated that 5-HT levels in the serum were not significantly different among the different groups after d 16 feeding or on different feeding days (*n* = 12, Fig. 1A and *n* = 24, Fig. 1C). No significant circadian rhythm had been found on the serum 5-HT levels among the groups at any given day (*n* = 24, *p* > 0.05) (Fig. 1B).

#### MT

**Figure 2:**
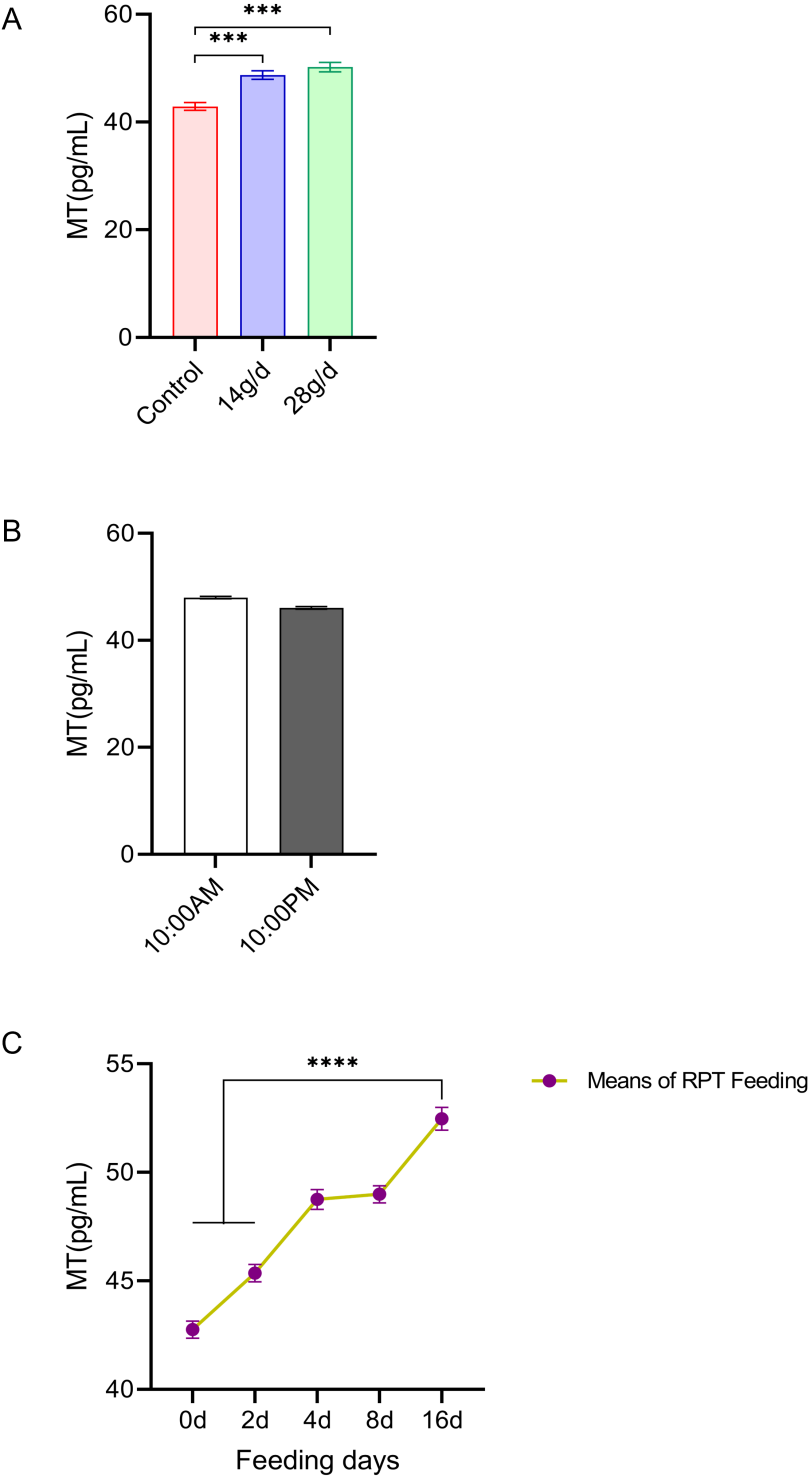
Effects of RPT feeding on MT levels in serum. (A) MT levels in different feeding groups; (B) MT levels in different time after RPT feeding; (C) changes of MT levels during RPT feeding days. The data are expressed as the mean ± SEM. One Way Analysis of variance (ANOVA) test was used to analyze the normality followed by student t-tests for comparison of the difference between the interested groups. For (B) only Student t-test was used. *** *p* < 0.001. **** *p* < 0.0001.

The results showed that RPT feeding groups at 14 g/d (48.73 ± 1.82 pg/mL) and 28 g/d (50.20 ± 2.01 pg/mL) had significantly increased serum MT levels compared to the control group (42.89 ± 1.62 pg/mL) after d 16 feeding (*n* = 12, *F* = 22.49, *p* < 0.001, Fig. 2A). The MT level at d 16 in RPT feeding group was 52.47 ± 2.55 pg/mL, which was significantly higher than that it was at the d 0 (42.75 ± 1.95 pg/mL) and d 2 (45.37 ± 1.86 pg/mL) (*n* = 12, *F* = 133.693, *p* < 0.0001, Fig. 2C). The MT levels at the 10:00 and 22:00 of a day had no significant difference among the groups (*p* > 0.05) (*n* = 24, Fig. 2B).

#### PRL

**Figure 3:**
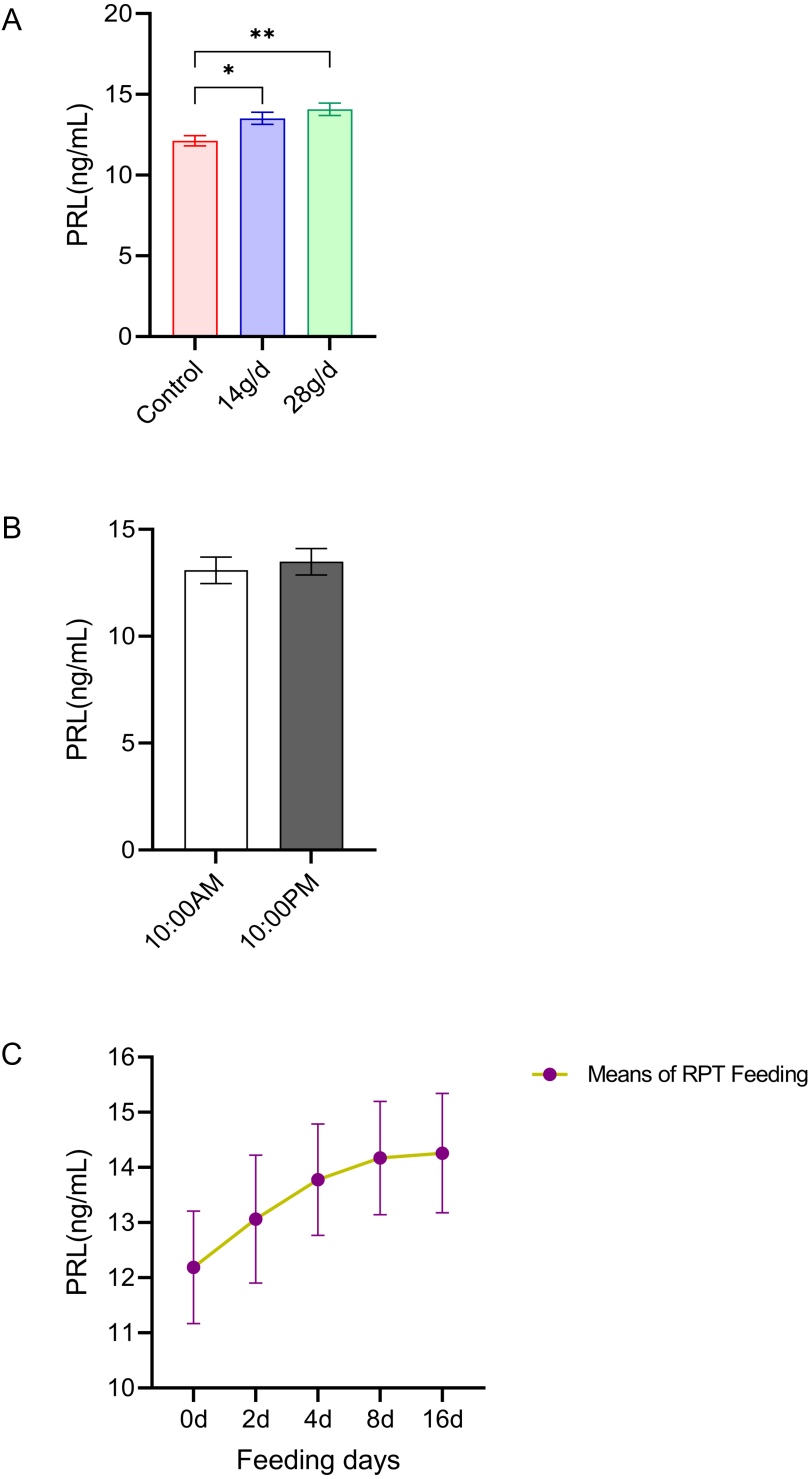
Effects of RPT feeding on PRL levels in serum. (A) PRL levels in different feeding groups; (B) PRL levels in different time after RPT feeding; (C) changes of PRL levels during RPT feeding days. The data are expressed as the mean ± SEM. One Way Analysis of variance (ANOVA) test was used to analyze the normality followed by student t-tests for comparison of the difference between the interested groups. For (B) only Student t-test was used.* *p* < 0.05, ** *p* < 0.01.

As shown in Fig. 3, the serum PRL levels in the 14 and 28 g/d RPT feeding groups were a 13.51±0.83 and 14.08±0.85 ng/mL, respectively, which were significantly higher than that in the control group (12.13 ± 0.72 ng/mL) after d 16 feeding (*n* = 12, *p* = 0.007, *F* = 7.845, Fig. 3A). However, the PRL levels were not significantly different among groups (*n* = 12, *p* > 0.05) (Fig. 3C). Also, no significant circadian rhythm had been detected on the PRL levels among groups (*n* = 24, *p* > 0.05) (Fig. 3B).

#### GH

**Figure 4:**
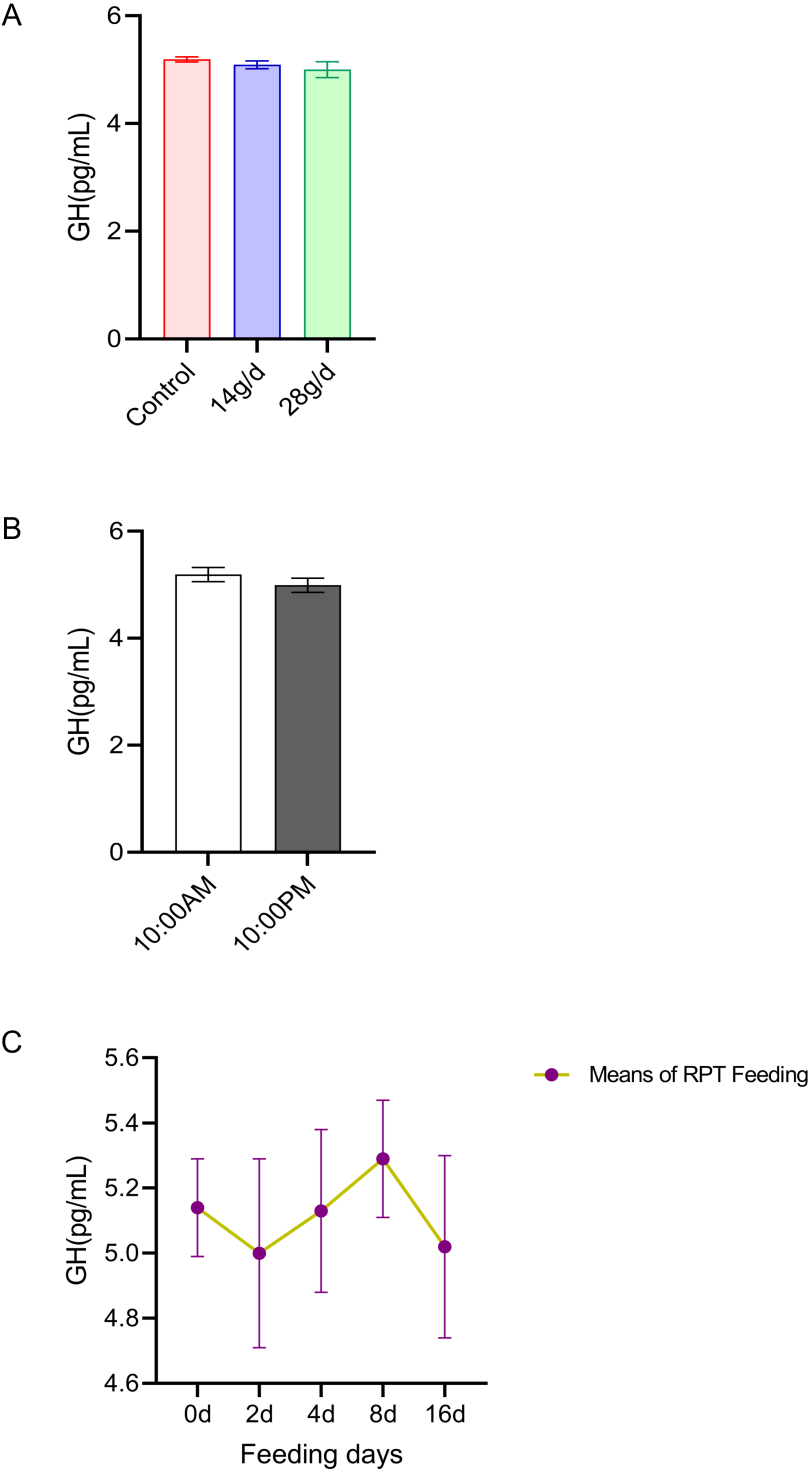
Effects of RPT feeding on GH levels in serum. (A) GH levels in different feeding groups; (B) GH levels in different time after RPT feeding; (C) changes of GH levels during RPT feeding days. The data are expressed as the mean ± SEM. One Way Analysis of variance (ANOVA) test was used to analyze the normality followed by student t-tests for comparison of the difference between the interested groups. For (B) only Student t-test was used.

The results indicated that GH levels in the serum were not significantly different among the different feeding groups after 16 d feeding or on different feeding days (*p* > 0.05) (*n* = 12, Fig. 4A and *n* = 24, Fig. 4C). The GH levels at 10:00 and 22:00 of different days (d 2, d 4, d 8, and d 16) had no significant difference among groups (*n* = 24, *p* > 0.05) (Fig. 4B).

#### IGF-1

**Figure 5:**
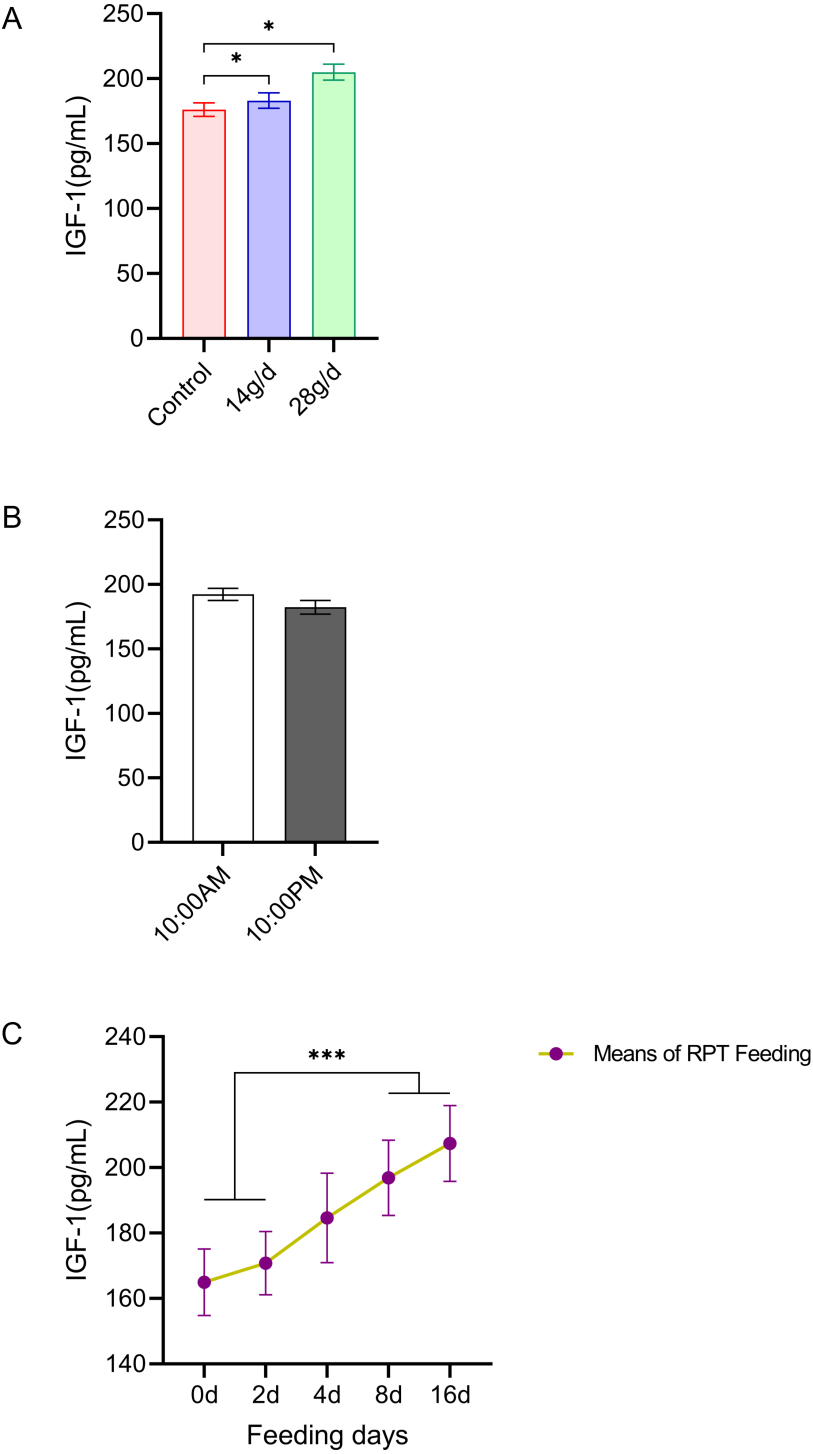
Effects of RPT feeding on IGF-1 levels in serum. (A) IGF-1 levels in different feeding groups; (B) IGF-1 levels in different time after RPT feeding; (C) changes of IGF-1 levels during RPT feeding days. The data are expressed as the mean ± SEM. One Way Analysis of variance (ANOVA) test was used to analyze the normality followed by student t-tests for comparison of the difference between the interested groups. For (B) only Student t-test was used.* *p* < 0.05, *** *p* < 0.001.

As shown in Fig. 5, the serum IGF-1 levels in the RPT feeding group at 14 g/d (183.06 ± 13.23 pg/mL) and 28g/d (204.98 ± 13.71 ng/mL) had significantly increased compared to the control group (176.18 ± 11.64 ng/mL) after d 16 feeding (*n* = 12, *p* = 0.011, *F* = 6.807, Fig. 5A). While the IGF-1 levels were 196.88 ± 11.52 ng/mL and 207.38 ± 11.61 ng/mL at the day 8 and day 16 after RPT feeding, respectively, which were significantly higher than that at d 0 (164.96 ± 10.17 ng/mL) and d 2 (170.81 ± 9.66 ng/mL) (*n* = 24, *p* < 0.001, *F* = 85.778, Fig. 5C). The IGF-1 levels at the times of 10:00 and 22:00 on different days (d 2, d 4, d 8, and d 16) had no significant difference among groups (*n* = 24, *p* > 0.05) (Fig. 5B).

## Discussion

Milk yield is affected by a variety of factors, such as breed, lactation stage, parity, illumination, disease, feed composition, and nutrition. The content and composition of amino acids obtained from fodder for the cows impact milk yield and milk components. For example, milk protein synthesis is highly dependent on the availability of essential amino acids in the mammary gland. Thus, rumen-protected amino acid (RPAA) supplementation in the diet not only decreases the supply of non-degraded protein feeds and increases the efficiency of amino acid utilization but also significantly enhances the total milk yield of lactating cows (Overton et al., 1996; Socha et al., 2005). A study found that adding 15 g/d rumen-protected methionine to the diet could increase milk yield, standard milk yield, and dry matter-corrected milk yield by 5, 6.2, and 6.5%, respectively (Schingoethe et al., 1988). Adding rumen-protected methionine to a diet based on corn, alfalfa silage, and soya bean cake increased milk yield by 6% (Illg, Sommerfeldt & Schingoethe, 1987). On other hand, adding RPT to the diet increased the small intestine tryptophan level, however, it had no significant effect on milk yield (Kollmann et al., 2008). Many studies have confirmed an important regulatory effect of tryptophan on the feed intake of livestock and poultry (Henry et al., 1992; Rosebrough, 1996; Coşkun et al., 2006; Cubero et al., 2006; Miao et al., 2019). A shortage of tryptophan will result in 5-HT deficiency of brain and lead to a sharp decline in animal feed intake (Haleem, 2017). In contrast, sufficient tryptophan in the diet increased 5-HT in the brain and improved animal feed intake (Henry et al., 1992; Rosebrough, 1996; Coşkun et al., 2006; Cubero et al., 2006; Miao et al., 2019). A controversial result was reported in hens that when tryptophan in the diet is beyond the normal physiological level, the animals’ food intake is inhibited (Haider et al., 1999; Coşkun et al., 2006). Downing, Joss & Scaramuzzi (1997) found that intravenous tryptophan infusion at 4.3 g/d has no significant effect on food intake in goats (Downing, Joss & Scaramuzzi, 1997). Our results showed that the addition of RPT had no significant effect on food intake in cows.

The content of milk protein is mainly affected by the composition, quantity, and balance of amino acids in the diet (Schwab et al., 1992). The content of milk protein is usually increased by adding dietary crude protein (CP) and rumen nondegradable protein (RNDP) to the diet (Martins et al., 2019; Zang et al., 2019). However, a variety of studies have indicated that CP and RNDP have little effect on milk protein. Thus, adding CP and RNDP to the diet is considered a waste of resources (Lamminen et al., 2019). In recent years, many studies have proved that supplementation with rumen-protected amino acids in the diet can significantly increase milk protein and casein in milk but has no significant effect on milk yield (Armentano, Swain & Ducharme, 1993; Rulquin & Vérité, 1996; Overton et al., 1996; Berthiaume et al., 2000; Socha et al., 2005). Therefore, providing the optimum ratio of amino acids is beneficial to improve the genetic potential of cow to synthesize milk proteins. The mammary gland absorbs methionine, histidine, phenylalanine, tyrosine, tryptophan, and other AA from the blood to synthesize milk protein. Especially, the essential amino acids, particularly tryptophan, exhibits high efficiency to cope with other amino acids for milk protein synthesis (Mepham, 1982).

Milk protein secretion is also regulated by hormones that act on the amino acid transport system and mammary gland blood flow. And then, improving the uptake and utilization of amino acids in the mammary gland increases milk protein levels (Rezaei et al., 2016). Tryptophan is a metabolically active amino acid that can be converted into serotonin (5-HT) and melatonin (MT) by respective enzymes (Kollmann et al., 2008).

5-HT temporarily inhibits brain activity, regulates emotion, which induces the animal into a quiet state, and then reduces energy consumption caused by a large amount of exercise (Gorka et al., 2019). As a result, it improves the growth performance of animals due to reduced maintenance requirements. 5-HT is also a feedback inhibitor of lactation and inhibits the secretion of hormones that promote mammary gland development and lactation in mammals (Wilde et al., 1995; Matsuda et al., 2004). Tryptophan hydroxylase (TPH) plays a vital role during the process of 5-HT synthesis and is mainly affected by tryptophan levels in the blood and brain (Bell, Abrams & Nutt, 2001). Under normal physiological conditions, the TPH level in the brain is higher than that in circulation. This indicates that TPH is in an unsaturated state (Fadda, 2000). Therefore, increasing tryptophan supplementation can further improve the synthesis of 5-HT in the brain (Fernstrom & Fernstrom, 2006).

Tryptophan is also a precursor of MT. Oral administration of 150–300 mg/kg tryptophan can significantly increase the levels of MT in the circulatory system of chickens and mice (Huether et al., 1992). Kollmann et al. (2008) supplied RPT in the daily ration to the heifers, which was shown to significantly increase their serum tryptophan and MT levels compared to the controls. Melatonin can stimulate the secretion of PRL and IGF-1 (Ma et al., 2012).

PRL improves the development of mammary glands prolacting secretory cells and initiates and maintains lactation (Huang, Maltecca & Khatib, 2008). PRL is mainly expressed in the prolactating cells of the anterior lobe of bovine adenohypophysis and binds to PRL receptors to activate the JAK2/STAT5 signaling pathway. This pathway acts on the milk protein gene promoter region and upregulates the expression of milk protein synthesis genes (Shu et al., 2020). Thus, the PRL level is positively correlated with milk-producing traits (Conneely, Mulac-Jericevic & Arnett-Mansfield, 2007).

IGF-1 is a single-chain polypeptide growth factor that is highly homologous to insulin. IGF-1 acts on target cells to cause anabolic changes by internal secretion, paracrine signaling, and autocrine signaling, which improves the synthesis of proteins and lipogenesis and gluconeogenesis at the transcriptional and translational levels to increase energy metabolism levels (Feng & Levine, 2010). It was reported that exogenous GH treatment can improve the milk yield of cows by increasing IGF-1 content. Studies also found that the galactagogue effect of long day length was closely related to the increase in IGF-1 secretion (Sevrin et al., 2020).

## Conclusion

Judging by the biological activities of tryptophan on milk production mentioned above, in the current study, RPT was used to increase the bioavailability of tryptophan in dairy cows. Cows are ruminant and have different digestive system from others. When the tryptophan is supplemented in the diet majority of the tryptophan will be degraded by the microorganisms in the rumen. We expected that the RPT would bypass the degradation process by the microorganisms and was transported to the mammary gland for milk protein synthesis. The results confirmed our expectation that RPT was a better method to deliver tryptophan to ruminants than the regular route. This was indicated by the significantly increased the milk protein percentage, milk protein yield, SNF and milk yield after RPT feeding in cows (*p* < 0.05). The mechanisms may mainly relate to the increased MT level in the serum with RPT feeding since melatonin is the major derivative of tryptophan and its synthesis is directly related to the availability of tryptophan. In turn, the upregulated melatonin level, then upregulates PRL and IGF-1 levels in circulation. PRL and IGF-1 are two major factors to promote milk protein synthesis and production. In conclusion, RPT feeding can be used to improve the quality and yield of milk in cows or other ruminates. The results are promising and require further studies in different ruminates.

##  Supplemental Information

10.7717/peerj.13831/supp-1 Supplemental Information 1 Raw Data

10.7717/peerj.13831/supp-2 Supplemental Information 2 Author Checklist-Full

## Additional Information and Declarations

### Data Availability

The following information was supplied regarding data availability: The raw measurements are available in the Supplemental File.
